# The Gaming Disorder Test and Gaming Disorder Scale for Adolescents: Translation and validation among Vietnamese young adults

**DOI:** 10.1186/s41155-024-00328-9

**Published:** 2024-10-18

**Authors:** Kamolthip Ruckwongpatr, Yu-Han Lee, Ngoc Dang Tran, Le An Pham, Mark D. Griffiths, Amir H. Pakpour, Cheng-Kuan Lin, Yu-Ting Huang, Jung-Sheng Chen, Sio-Meng Lei, Chung-Ying Lin

**Affiliations:** 1 Institute of Allied Health Sciences, College of Medicine, National Cheng Kung University, Tainan, 701 Taiwan; 2 Department of Public Health, College of Medicine, National Cheng Kung University, Tainan, 701 Taiwan; 3 Department of Environmental and Occupational Health, University of Medicine and Pharmacy at Ho Chi Minh City, Ho Chi Minh City, Vietnam; 4 Center of Family Medicine, University of Medicine and Pharmacy at Ho Chi Minh City, Ho Chi Minh City, Vietnam; 5 International Gaming Research Unit, Psychology Department, Nottingham Trent University, Nottingham, NG1 4FQ UK; 6 Department of Nursing, School of Health and Welfare, Jönköping University, 55318 Jönköping, Sweden; 7 Institute of Public Health, National Yang Ming Chiao Tung University, Taipei, 112 Taiwan; 8 Department of Medical Research, E-Da Hospital, I-Shou University, Kaohsiung, 82445 Taiwan; 9 Department of Psychiatry, E-Da Hospital, I-Shou University, No. 1, Yida Rd., Yanchao Dist., Kaohsiung, 82445 Taiwan; 10 School of Medicine, I-Shou University, Kaohsiung, 82445 Taiwan; 11 Biostatistics Consulting Center, College of Medicine, National Cheng Kung University Hospital, National Cheng Kung University, Tainan, 701 Taiwan; 12 Department of Occupational Therapy, College of Medicine, National Cheng Kung University, Tainan, 701 Taiwan; 13 Departments of Occupational Therapy and Public Health, and Biostatistics Consulting Center, Institute of Allied Health Sciences, College of Medicine, National Cheng Kung University Hospital, National Cheng Kung University, 1 University Rd, Tainan, 701401 Taiwan

**Keywords:** Emerging adults, Factor analysis, Gaming disorder, Problematic gaming, Psychometric testing

## Abstract

**Background:**

Previous studies have shown that the Gaming Disorder Test (GDT) and Gaming Disorder Scale for Adolescents (GADIS-A) have promising validity and reliability when assessing symptoms of gaming disorder among young adults. However, validity and reliability properties of the two scales have not been established among a Vietnamese population.

**Objective:**

The present study translated the GDT and GADIS-A into Vietnamese and examined their factor structures, measurement invariance, convergent validity, concurrent validity, and divergent validity among university students.

**Methods:**

A total of 610 young adults (mean age = 21.09 years; 63.4% females) were recruited using convenience sampling and who completed a paper-and-pencil survey between April and June 2023. All participants completed a demographic questionnaire, GDT, GADIS-A, and six standardized scales related to gaming disorder, social media addiction, smartphone addiction, and psychological distress. Confirmatory factor analysis (CFA), internal consistency testing, and Pearson’s correlations were performed.

**Results:**

CFA showed that the GDT had a one-factor structure and the GADIS-A had a two-factor structure. The internal consistency was excellent for both scales among this population. Moreover, both GDT and GADIS-A showed convergent, concurrent, and divergent validity with other standardized scales.

**Conclusion:**

The Vietnamese versions of the GDT and GADIS-A have good psychometrics, which may be utilized in future research regarding gaming disorder among Vietnamese populations.

## Introduction

The increasing popularity of online gaming has resulted in various concerns related to problematic gaming use, including negative impact on the daily lives and poor health among a minority of gamers (Billieux et al., 2015). Previous meta-analyses reported global prevalence of gaming disorder (GD) to range from 1.96% to 4.6% with higher rates among males compared to females (Fam, 2018; Kim et al., 2022; Stevens et al., 2021). Previous empirical studies have also reported that gamers in the Southeast Asia region (including Vietnam where the present study was carried out) have higher rates of GD (10.1%) than other regions (Chia et al., 2020). However, those working in the area have noted that the prevalence of GD among gamers in Asian countries is needed to confirm the findings (Mihara et al., 2017). Moreover, scholars in the field have strongly encouraged the development and validation of standardized instruments to screen and diagnose GD to produce more reliable and comparable statistics regarding the prevalence of GD in Asian countries (Mihara et al., 2017). More importantly, such prevalence data using validated instruments would enhance the investigation and treatment in problematic gaming among those in Asian countries (Chia et al., 2020).

GD has formally been described as a behavioral addiction by the World Health Organization (WHO) in the International Classification of Diseases, 11th Revision (ICD-11) (Kim et al., 2022). The American Psychiatric Association (APA) has also included internet gaming disorder (IGD) as a condition for further research in the fifth edition of Diagnostic and Statistical Manual of Mental Disorders (DSM-5) (American Psychiatric Association, 2013). The WHO framework for GD includes diagnostic concepts such as functional impairment while gaming, while the APA framework for IGD includes more traditional addiction criteria such as withdrawal and tolerance (as well as functional impairment) (Jo et al., 2019). Although there are some differences in diagnostic concepts regarding GD and IGD, empirical evidence has shown that the diagnostic concepts from the WHO framework are associated with pathological aspects for GD, suggesting the robustness of using ICD-11 diagnostic criteria to screen for GD symptoms (Jo et al., 2019; Montag et al., 2019). Moreover, the WHO framework adopts a time period of a year (i.e., the same period defined by the DSM-5) to further diagnose GD (Pontes et al., 2021). The literature also suggests future study to develop and validate additional instruments based on the WHO diagnostic framework to improve identification and diagnosis of GD (Carragher et al., 2022).

The ICD-11 framework defines GD as persistent or recurrent online and/or offline gaming behavior within the past 12 months and indicated by three main diagnostic criteria: (i) impaired control over the gaming activity; (ii) increasing priority to the extent that gaming takes precedence over other interests and daily activities; and (iii) continuation or escalation of gaming activity despite the occurrence of negative consequences (Montag et al., 2019). The ICD-11 diagnostic criteria further suggest other GD clinical symptoms which relate to the impact of individuals’ daily living, including their personal, family, social, education, occupational, and/or other significant area of functioning due to their gaming behavior (Montag et al., 2019).

Based on the diagnostic criteria proposed by the WHO in the ICD-11, the Gaming Disorder Test (GDT) and the Gaming Disorder Scale for Adolescents (GADIS-A) were both developed (Paschke et al., 2020; Pontes et al., 2021). Psychometric evidence regarding the two instruments further shows that they have strong psychometric properties in the assessment of gaming severity (Chen et al., 2023).

The GDT was developed based on the ICD-11 diagnostic framework to assess GD among adults (Pontes et al., 2021). The GDT is a brief screening instrument with unidimensional factor structure and has been found to be a robust measure that assesses all three main ICD-11 diagnostic criteria and significant impairments due to gaming (Karhulahti et al., 2023). Similar to the GDT, the GADIS-A was also developed using the ICD-11 criteria but assessed GD among children and adolescents (Paschke et al., 2020). The GADIS-A has a two-dimensional factor structure, and its items assess all three main ICD-11 diagnostic criteria (Paschke et al., 2020). Notably, the GADIS-A also has additional items which assess the duration of GD symptoms according to the ICD-11 (Paschke et al., 2020). Additionally, previous studies have shown that GADIS-A has high internal consistency across gender and young adults (Chen et al., 2023; Ghazi et al., 2024; Wu et al., 2023). Therefore, there is literature suggesting that the two instruments (i.e., GDT and GADIS-A) should be cross-validated among different populations to increase their external validity and to provide further psychometric evidence of two scales given that they were both developed using the ICD-11 criteria (Pontes et al., 2021; Wu et al., 2023).

To the best of the present authors’ knowledge, only two instruments that assess GD have been translated and validated among Vietnamese people (i.e., the Online Game Addiction Scale and the 20-item Internet Gaming Disorder Test [IGDT-20]) (Cuong et al., 2021; Son et al., 2013). However, both of these instruments were developed prior to GD being included in the ICD-11 and therefore have different criteria. Therefore, there are currently no psychometric instruments based on the WHO diagnostic GD framework available for Vietnamese individuals. In order to facilitate the screening efficiency for GD diagnosis, GD instruments based on ICD-11 diagnostic criteria (e.g., GDT and GADIS-A) are needed. This would allow mental health professionals and researchers in Vietnam to use these instruments to assess and carry out empirical research examining GD severity among Vietnamese youth (including emerging adults).

Therefore, the aim of the present study was to evaluate the translated Vietnamese versions of the GDT and GADIS-11. The study also had four hypotheses. These were that: (i) the Vietnamese version of GDT would have a one-factor structure and the GADIS-A would have a two-factor structure with satisfactory fit indices as shown through confirmatory factor analysis (H_1_); (ii) measurement invariance would be supported across gender (male vs. female) and gaming time (< 2 h daily vs. ≥ 2 h daily) for both GDT and GADIS-A (H_2_); (iii) both the GDT and GADIS-A would have adequate convergent validity with each other and with an external standardized scale that assesses the risk for developing GD (H_3_); and (iv) both the GDT and GADIS-A would have adequate concurrent and divergent validity with standardized scales that assess the risk for developing other types of online behavioral addiction (i.e., social media addiction and smartphone addiction) and psychological distress (H_4_).

## Methods

### Participants and procedure

The present study was cross-sectional, and the data were collected using convenience sampling with participants who completed a paper-and-pencil survey. A total of 610 Vietnamese young adults (63.4% females) from three departments were recruited between April and June 2023: pharmacy (35.7%), medicine (34.4%), and nursing (29.8%). The sample had a mean age of 21.09 years (SD = 1.74; age range 19 to 32 years).

Evidence has shown that adolescence is a period of transition in the development of physical and psychological characteristics from childhood to adulthood (Nazari et al., 2022). Therefore, young adulthood can be seen as an extension or a prolonged age of adolescence. Moreover, a prior study noted that emerging adulthood is actually a period of late adolescence through to individuals in their twenties (i.e., age 18–25 years) (Arnett, 2000). Moreover, research evidence indicates that prevalence of GD among Asian young adults (especially, university students) is high compared to other world regions particularly because of their use of Wi-Fi-enabled devices (e.g., smartphones, tablets) (Chia et al., 2020). Additionally, emerging adults have been identified as a vulnerable group for GD (Samaranada et al., 2023). Therefore, one of the easiest ways to access this demographic is through convenience sampling of university students.

Before data collection, the study’s procedures were approved by the Board of Ethics in Biomedical Research in the University of Medicine and Pharmacy at Ho Chi Minh City (Number: 1080/UMP-BOARD). All participants were approached during their recess period between classes with the assistance of the faculty members affiliated to the: Faculty of Medicine, Faculty of Pharmacy, and Faculty of Nursing and Medical Technology (University of Medicine and Pharmacy at Ho Chi Minh City). The participants were informed of the study’s purpose and given the survey if they voluntarily agreed to participate and provided written informed consent. The survey included demographic information and six standardized psychometric scales: GDT, GADIS-A, Bergen Social Media Addiction Scale (BSMAS), Smartphone Application-Based Addiction Scale (SABAS), Internet Gaming Disorder Scale-Short Form (IGDS9-SF), and Depression Anxiety and Stress Scale -21 (DASS-21). The survey took approximately 15 to 20 min to complete all the questions. The inclusion criteria were: (i) being aged ≥ 18 years; (ii) being able to understand and read the Vietnamese language; and (iii) being enrolled at universities in Vietnam.

### Translation procedure

After obtaining permission from the developers of GDT and GADIS-A, they were both translated into Vietnamese and underwent a process of cross-cultural adaptation (Beaton et al., 2000; Paschke et al., 2020; Pontes et al., 2021). First, GDT and GADIS-A were translated from English to Vietnamese language by two bilingual native individuals. Then, both forward translations were synthesized into a final forward translated version through discussion between the aforementioned translators. Then, the final forward translation was translated back from Vietnamese to English language by two different independent Vietnamese translators who were blind to the original version (backward translation). The final versions of the scales were agreed by three experts who reviewed all items (i.e., two forward translations, one final forward translation, two backward translations, and original versions) to produce the final versions of the GDT and GADIS-A. More specifically, an *Excel* file was used to compile all the GDT and GADIS-A versions generated during the translation procedure. The three experts were instructed to read the file and to evaluate if the final forward translation accurately reflected the original version. If they found any discrepancies, they were instructed to read all the other versions (i.e., preliminary forward translations and back translations) to provide suggestions on modifications. The three experts did the evaluations separately without any interaction between each other. Finally, all three experts fully agreed with the final versions of the GDT and GADIS-A and did not suggest any modifications regarding cultural adaptation.

### Measures

#### Demographic information

Participants were asked to provide information regarding their age, gender, education degree, study major, and further questions regarding their internet use in the past week: (1) daily hours spent using social media; (2) daily hours spent gaming; and (3) daily hours spent on online learning.

#### Gaming Disorder Test (GDT)

The GDT was used to assess GD and the individual’s experience of online/offline gaming activities and devices used for gaming (e.g., computer, consoles, smartphones, etc.) in the past year (Pontes et al., 2021). The GDT comprises four items relating to the diagnostic criteria for GD in the ICD-11, and each item is responded on a five-point Likert scale from 1 (*never*) to 5 (*very often*) (Pontes et al., 2021). A sample item in the GDT is *“I have continued gaming despite the occurrence of negative consequences.”* Total scores are calculated by summing the items, and a higher total GDT score reflects higher severity of GD (Pontes et al., 2021). The GDT has been translated and validated in a number of languages including English (Cronbach's α = 0.85) (Pontes et al., 2021), Chinese (Cronbach's α = 0.90) (Wu et al., 2023), Malaysian (Cronbach's α = 0.86) (Ghazi et al., 2024), Turkish (Cronbach's α = 0.88) (Evren et al., 2020), and Thai (McDonald’s ω = 0.70) (Saffari et al., 2024). The Cronbach's α in the present study was 0.85.

#### Gaming Disorder Scale for Adolescents (GADIS-A)

The GADIS-A was used to assess GD and the individual’s experience of online/offline gaming activities and devices used for gaming (e.g., computer, consoles, smartphones, etc.) in the past year (Paschke et al., 2020). The scale comprises 10 items relating to the diagnostic criteria for GD in the ICD-11 and has three subscales: (i) cognitive behavior symptoms (CBS); (ii) negative consequences (NC); and (iii) frequency of GD symptoms (Paschke et al., 2020). The first nine items refer to CBS subscale (Items: 1, 2, 4, and 5), and NC subscale (Items: 3, 6, 7, 8, and 9). Each item is responded on a five-point Likert scale from 0 (*strongly disagree*) and 4 (*strongly agree*). Item 10 refers to the frequency of GD symptoms subscale and comprises four responses: 0 (*not at all*), 1 (*only on single days*), 2 (*during longer periods*), 3 (*almost daily*). A sample item of the CBS subscale is *“I often cannot stop gaming even though it would be sensible to do so or, for example, my parents have told me to stop.”* A sample item of NC subscale is *“Due to gaming, I risk losing important contacts or have lost them already. This includes contacts with partners, friends, acquaintances or family.”* The frequency of GD symptoms subscale item is *“In the past year, how often did you experience the conflicts or difficulties described in the statements 1 to 9 due to gaming? Did this only occur on single days, during longer periods of several weeks to months, or was it almost daily?”* Total scores are calculated by summing each subscale, and a higher score than cut-off points (CBS subscale score > 5; and NC subscale score > 9) reflects higher severity on each subscale (Paschke et al., 2020; Nazari et al., 2022). The frequency of symptoms subscale (Item 10) has a time criterion score of 2 (*during longer periods*) or 3 (*almost daily*) and this item is not included for GD structure in the GADIS-A (Paschke et al., 2020). The GADIS-A has been translated and validated in a number of languages including German (Cronbach's α = 0.87–0.91) (Paschke et al., 2020), Chinese (Cronbach's α = 0.89–0.94) (Wu et al., 2023), Malaysian (Cronbach's α = 0.90–0.95) (Ghazi et al., 2024), Thai (McDonald’s ω = 0.82–0.91) (Saffari et al., 2024), and Russian (Cronbach's α = 0.82–0.85) (Nazari et al., 2022). The Cronbach's α was 0.87–0.93 in the present study.

#### Bergen Social Media Addiction Scale (BSMAS)

The BSMAS was used to assess the risk of social media addiction over the past year (Andreassen et al., 2016). The BSMAS comprises six items based on the ‘addiction components model’ (Griffiths, 2005), and each item is responded on a five-point Likert scale from 1 (*very rarely*) to 5 (*very often*). A sample item is *“You have tried to cut down on the use of social media without success.”* Total scores are calculated by summing the items, and a higher total score reflects a greater risk of social media addiction (Andreassen et al., 2016). The BSMAS has been translated and validated in a number of languages including Norwegian (Cronbach's α = 0.88) (Andreassen et al., 2016), Chinese (Cronbach's α = 0.82–0.85) (Leung et al., 2020), Persian (Cronbach's α = 0.86) (Lin et al., 2017), Italian (Cronbach's α = 0.88) (Monacis et al., 2017), and Vietnamese (Cronbach's α = 0.81) (Doan et al., 2022). The Cronbach's α was 0.83 in the present study.

#### Smartphone Application-Based Addiction Scale (SABAS)

The SABAS was used to assess the risk of smartphone addiction over the past year (Csibi et al., 2018). The SABAS comprises six items based on the ‘addiction components model’ (Griffiths, 2005), and each item is responded to on a six-point Likert scale from 1 (*strongly disagree*) to 6 (*strongly agree*). A sample item is *“If I cannot use my smartphone when I feel like, I feel sad, moody, or irritable.”* Total scores are calculated by summing the items, and a higher total score reflects a greater risk of smartphone addiction (Leung et al., 2020). The SABAS has been translated and validated in a number of languages including English (Cronbach's α = 0.81) (Csibi et al., 2018), Chinese (Cronbach's α = 0.78–0.79) (Leung et al., 2020), and Indonesian (Cronbach's α = 0.74) (Nurmala et al., 2022). The Cronbach's α was 0.82 in the present study.

#### Internet Gaming Disorder Scale-Short Form (IGDS9-SF)

The IGDS9-SF was used to assess internet gaming disorder over the past year (Pontes and Griffiths, 2015). The IGDS9-SF comprises nine items based on the DSM-5 criteria for IGD (American Psychiatric Association, 2013). Each item is responded to on a five-point Likert scale from 1 (*never*) to 5 (*very often*). A sample item is *“Have you lost interests in previous hobbies because of your engagement with gaming?”* Total scores are calculated by summing the items, and a higher score reflects greater severity of IGD (Pontes and Griffiths, 2015). The IGDS9-SF has been translated and validated in a number of languages including English (Cronbach's α = 0.87) (Pontes and Griffiths, 2015), Chinese (Cronbach's α = 0.94) (Wu et al., 2023), Persian (Cronbach's α = 0.90) (Wu et al., 2017), and Vietnamese (Cronbach's α = 0.87) (Stevanović et al., 2020). The Cronbach's α was 0.92 in the present study.

#### Depression Anxiety and Stress Scale -21 (DASS-21)

The DASS-21 was used to assess psychological distress over the previous seven days (Henry & Crawford, 2005; Le et al., 2017; Wu et al., 2023). The DASS-21 comprises 21 items across three domains (i.e., depression, anxiety, and stress), each with seven items. Each item is responded to on a four-point Likert scale from 0 (*Did not apply to me at all – Never*) to 3 (*Applied to me very much, or most of the time – Almost always)* (Le et al., 2017). A sample item in the depression subscale is *“I found it difficult to work up the initiative to do things”.* A sample item in the anxiety subscale is *“I experienced trembling (e.g., in the hands)”.* A sample item in the stress subscale is *“I found myself getting agitated.”* Final scores are calculated by summing the items in each domain (or the total) (Wu et al., 2023). Higher total score reflects greater psychological distress, and higher scores on each domain reflect higher severity of depression, anxiety, and stress. The DASS-21 has been translated and validated in many languages including Chinese (Cronbach's α = 0.83–0.94) (Wu et al., 2023), English (Cronbach's α = 0.82–0.93) (Henry & Crawford, 2005), and Vietnamese (Cronbach's α = 0.74–0.91) (Le et al., 2017). The Cronbach's α was 0.94 in the present study.

### Statistical analysis

The present study initially adopted descriptive statistics to examine the participants’ demographic information, and mean scores of on the GDT, GADIS-A, BSMAS, SABAS, IGDS9-SF and DASS-21. Moreover, there was an examination of the item properties of GDT and GADIS-A using factor loadings (derived from confirmatory factory analysis [CFA]) and item rest correlation. Values of 0.4 or higher of both factor loadings and item-rest correlation indicates measure acceptability (Hair et al., 2018). Subsequently, CFA, using diagonally weighted least squares (DWLS) estimation, was used to examine the factor structure of GDT (one factor) and GADIS-A (two factors). The χ^2^ test, standardized root mean square residual (SRMR), root mean square error of approximation (RMSEA), Tucker-Lewis index (TLI), and comparative fit index (CFI) were employed to examine the model fit indices. The criteria utilized to evaluate model fit in CFA included: nonsignificant χ^2^, CFI > 0.95, TLI > 0.95, RMSEA < 0.05 and SRMR < 0.05 (Schermelleh-Engel et al., 2003). Additionally, Cronbach’s alpha and McDonald’s omega were used to examine internal consistency of the GDT and GADIS-A items. Values of 0.7 or higher of both Cronbach’s alpha and McDonald’s omega indicate acceptability of the measure (Kalkbrenner, 2023; Nunnally, 1978).

More importantly, the present study adopted multi-group CFA (MGCFA) to ensure equivalence of the GDT and GADIS-A structures across gender (females vs. males) and gaming time (< 2 h daily vs. ≥ 2 h daily) by examining and comparing between three nested models. According to previous studies, the GDT and GADIS-A were equivalent across the number of hours spent gaming (< 2 h daily vs. ≥ 2 h daily) (Chen et al., 2023; Ghazi et al., 2024). Therefore, the present study adopted two specific time thresholds (< 2 h daily vs. ≥ 2 h daily) to examine the equivalence of GDT and GADIS-A structures in the Vietnamese versions. The three nested models comprised: (M0) configural invariance (testing equivalence of the factor structure); (M1) metric invariance (testing equivalence of factor loadings constrained across subgroups); and (M2) scalar invariance (testing equivalence of factor loadings with item intercepts constrained across subgroups). Establishing the measurement invariance across subgroups, every two nested models are compared. The measurement invariance is supported when the following criteria are met: non-significant *χ* ^2^ difference test, and changes of ΔCFI > -0.01, ΔRMSEA < 0.015, and ΔSRMR < 0.03 (for factor loading) or < 0.01 (for item threshold) (Chen, 2007).

To ensure convergent and divergent validity, Pearson correlations were used to examine GDT and GADIS-A by comparing their correlations with scores on the BSMAS, SABAS, IGDS9-SF, and DASS-21. JASP 0.17.2.1 was used for all the statistical analysis (JASP Team, 2023).

## Results

As shown in Table 1, on average, the participants engaged in 2.87 h of social media use daily (SD = 1.85), 1.07 h of gaming daily (SD = 1.49), and 1.59 h of online learning daily (SD = 2.10). The participants’ gaming time was categorized into two groups (< 2 h daily vs. ≥ 2 h daily). A total of 179 participants engaged in gaming ≥ 2 h daily (29.3%). Additionally, the mean score on the GDT was 6.34 (out of 20; SD = 2.85). The mean score on the GADIS-A was 5.8 (out of 36; SD = 5.90) for the total score, 2.87 (out of 16; SD = 3.12) for CBS subscale, and 2.93 (out of 20; SD = 3.26) for NC subscale. The mean score on the BSMAS was 14.20 (out of 30; SD = 4.60). The mean score on the SABAS was 17.40 (out of 36; SD = 5.40). The mean score on the IGDS9-SF was 13.36 (out of 45; SD = 5.39). The mean score on the DASS-21 was 27.7 (out of 63; SD = 20.80).

**Table 1:** Characteristics of the present sample (*N* = 610)

Variable	Mean (SD) or n (%)
**Age in years**	21.09 (1.74)
**Sex (female)**	387 (63.40)^a^
**Major**	
Pharmacy	218 (35.70)
Medicine	210 (34.40)
Nursing	182 (29.80)
**Internet use (hours per day)**	
Time spent on using social media	2.87 (1.85)
Time spent on gaming	1.07 (1.49)
Time spent on online learning	1.59 (2.10)
**Gaming time ≧2 h per day**	179 (29.30)
**Scales of problematic internet use**	
GDT	6.34 (2.85)
GADIS-A(T)	5.80 (5.90)
GADIS-A(CBS)	2.87 (3.12)
GADIS-A(NC)	2.93 (3.26)
BSMAS	14.20 (4.60)
SABAS	17.40 (5.40)
IGDS9-SF	13.36 (5.39)
**Scale for psychological distress**	
DASS-21	27.70(20.80)

*GDT* Gaming Disorder Test, *GADIS-A(T)* Gaming Disorder Scale for Adolescents (Total score), *GADIS-A(CBS)* Gaming Disorder Scale for Adolescents (cognitive behavioral symptoms subscale score), *GADIS-A(NC)* Gaming Disorder Scale for Adolescents (negative consequences subscale score), *BSMAS* Bergen Social Media Addiction Scale, *SABAS* Smartphone Application Based Addiction Scale, *IGDS9-SF* Internet Gaming Disorder Scale-Short Form, *DASS-21* Depression Anxiety Stress Scale–21

^a^Three participants did not report their gender information

As shown in Table 2, the mean and SD values were low on each item of the GDT and GADIS-A. Moreover, the factor loading values were higher than 0.4 for all items on the GDT (0.63–0.90) and GADIS-A (0.78–0.86 for CBS subscale; 0.74–0.85 for NC subscale). The values of the item-rest correlation were also higher than 0.4 for all items of GDT (0.61–0.74) and GADIS-A (0.71–0.79 for CBS subscale; 0.71–0.79 for NC subscale).

**Table 2:** Item contents and item properties of the Gaming Disorder Test (GDT) and Gaming Disorder Scale for Adolescents (GADIS-A)

	Item content	Mean (SD)	Factor loading	Item-rest correlation
**GDT**				
GDT_I1	Difficulty in controlling gaming activity	1.81 (0.96)	0.83	0.68
GDT_I2	Increased priority to gaming over other activities	1.64 (0.88)	0.77	0.73
GDT_I3	Continued gaming despite negative consequences	1.52 (0.86)	0.90	0.74
GDT_I4	Significant problems due to gaming	1.36 (0.73)	0.63	0.61
**GADIS-A**				
**CBS subscale**				
GADIS-A_I1	More frequent on gaming	0.97 (1.04)	0.78	0.71
GADIS-A_I2	Unable to stop gaming	0.77 (0.86)	0.81	0.78
GADIS-A_I4	Neglect daily duties due to gaming	0.57 (0.73)	0.86	0.79
GADIS-A_I5	Continue gaming despite causing stress	0.56 (0.72)	0.80	0.77
**NC subscale**				
GADIS-A_I3	No other interests except for gaming	0.57 (0.74)	0.79	0.75
GADIS-A_I6	Continue gaming although harming school/apprenticeship/job performance	0.72 (0.86)	0.85	0.79
GADIS-A_I7	Neglect health due to gaming	0.54 (0.77)	0.74	0.71
GADIS-A_I8	Lose important contacts due to gaming	0.49 (0.69)	0.81	0.73
GADIS-A_I9	Have disadvantages at school/apprenticeship/job due to gaming	0.62 (0.81)	0.83	0.79

*CBS* Cognitive behavioral symptoms, *NC* Negative consequences

As shown in Table 3, a CFA of the GDT showed excellent model fit with a one-factor structure among Vietnamese university students (non-significant χ^2^(df) = 0.52 (1); CFI = 1.000; TLI = 1.000; RMSEA = 0.000; SRMR = 0.015). Similarly, a CFA of the GADIS-A showed excellent model fit with a two-factor structure among Vietnamese university students (nonsignificant χ^2^(df) = 19.64 (22); CFI = 1.000; TLI = 1.000; RMSEA = 0.000; SRMR = 0.038). Additionally, no modification indices were used for either the GDT or GADIS-A to achieve excellent model fit. Moreover, the internal consistency values were higher than 0.7 for both the GDT (McDonald’s ω = 0.90 and Cronbach's α = 0.85) and the GADIS-A (McDonald’s ω = 0.96 for total score; 0.89 for CBS subscale score; 0.92 for NC subscale score, and Cronbach's α = 0.93 for total score; 0.87 for CBS subscale score; 0.89 for NC subscale score).

**Table 3:** Confirmatory factor analysis and internal consistency of the Gaming Disorder Test (GDT) and Gaming Disorder Scale for Adolescents (GADIS-A)

	GDT	GADIS-A
McDonald’s ω	0.90	0.96 [entire GADIS-A]; 0.89 [CBS]; 0.92 [NC]
Cronbach's α	0.85	0.93 [entire GADIS-A]; 0.87 [CBS]; 0.89 [NC]
χ^2^(df)	0.52 (1)	19.64 (22)
CFI	1.000	1.000
TLI	1.000	1.000
RMSEA	0.000	0.000
SRMR	0.015	0.038

*CFI* Comparative fit index, *TLI* Tucker-Lewis index, *RMSEA* Root mean square error of approximation, *SRMR* Standardized root mean square residual

*CBS* Cognitive behavioral symptoms, *NC* Negative consequences

As shown in Table 4, the MGCFA of the GDT across gender (females vs. males) showed non-significant χ^2^ differences and the changes of CFI, RMSEA and SRMR (in model M2 vs. M1) which indicated measurement invariance (*p*-value = 0.765, ∆CFI = 0.003, ∆RMSEA = -0.017, ∆SRMR = -0.008). However, significant χ^2^ differences were found and the change of RMSEA and SRMR (in model M1 vs. M0) were slightly higher that the values of criteria for measurement invariance (*p*-value = 0.024, ∆CFI = -0.008, ∆RMSEA = 0.047, ∆SRMR = 0.038). Additionally, the MGCFA of the GADIS-A across gender (females vs. males) showed non-significant *χ* ^2^ differences and the changes of CFI, RMSEA, SRMR all indicated measurement invariance (M1 vs. M0; *p*-value = 0.413, ∆CFI = 0.000, ∆RMSEA = 0.000, ∆SRMR = 0.005 and M2 vs. M1; *p*-value = 0.253, ∆CFI = 0.000, ∆RMSEA = 0.000, ∆SRMR = -0.002) between all two nested models.

**Table 4:** Measurement invariance of the Gaming Disorder Test (GDT) and Gaming Disorder Scale for Adolescents (GADIS-A) across gender (males vs. females) and time spent on gaming (< 2 h daily vs. ≥ 2 h daily)

	GDT			GADIS-A		
**Across gender**	M0	M1vs.M0	M2vs.M1	M0	M1vs.M0	M2 vs.M1
χ^2^(df)/ p-value	2.170 (4)/0.704	–	–	51.023 (54)/0.59	–	–
Δ χ^2^(Δdf)/ p-value	–	9.462 (3)/0.024	1.152 (3)/0.765	–	8.217 (8)/0.413	10.17 (8)/0.253
CFI	1.000	–	–	1.000	–	–
ΔCFI	–	-0.008	**0.003**	–	**0.000**	**0.000**
RMSEA	0.000	–	–	0	–	–
ΔRMSEA	–	0.047	**-0.017**	–	**0.000**	**0.000**
SRMR	0.003	–	–	0.056	–	–
ΔSRMR	–	0.038	**-0.008**	–	**0.005**	**-0.002**
**Across time spent gaming**						
χ^2^(df)/ p-value	1.094 (2)/0.579	–	–	31.498 (44)/0.921	–	–
Δ χ^2^(Δdf) / p-value	–	6.36 (3)/0.095	2.824 (3)/0.420	–	5.458 (7)/0.604	17.505 (7)/0.014
CFI	1.000	–	–	1.000	–	–
ΔCFI	–	**-0.005**	**0.000**	–	**0.000**	**0.000**
RMSEA	0.000	–	–	0.000	–	–
ΔRMSEA	–	**0.040**	**-0.009**	–	**0.000**	**0.000**
SRMR	0.021	–	–	0.042	–	–
ΔSRMR	–	0.028	**-0.006**	–	**0.004**	**0.002**

Bold values indicate invariance: ΔCFI > -0.01; ΔRMSEA < 0.015; ΔSRMR < 0.03 (for factor loading) or < 0.01 (for item threshold)

*M0* configural model, *M1* Model with loadings constrained equal, *M2* Model with loadings and thresholds constrained equal

*CFI* Comparative fit index, *RMSEA* Root mean square error of approximation, *SRMR* Standardized root mean square residual

The MGCFA of the GDT across gaming time (< 2 h daily vs. ≥ 2 h daily) showed non-significant χ^2^ differences and the changes of CFI, RMSEA, SRMR (in model M2 vs. M1) all indicated measurement equivalence (*p*-value = 0.420, ∆CFI = 0.000, ∆RMSEA = -0.009, ∆SRMR = -0.006). However, non-significant χ^2^ differences were found and the changes in CFI, RMSEA, SRMR (in model M1 vs. M0) were slightly higher than the values of criteria for measurement invariance (*p*-value = 0.095, ∆CFI = -0.005, ∆RMSEA = 0.040, ∆SRMR = 0.028). The MGCFA of the GADIS-A across gaming time (< 2 h daily vs. ≥ 2 h daily) showed non-significant χ^2^ differences and the changes of CFI, RMSEA, SRMR (in model M1 vs. M0) all indicated measurement equivalence (*p*-value = 0.604, ∆CFI = 0.000, ∆RMSEA = 0.000, ∆SRMR = 0.004). Additionally, the MGCFA of the GADIS-A across gaming time (< 2 h daily vs. ≥ 2 h daily) showed significant χ^2^ differences and the changes in CFI, RMSEA, SRMR (in Model M2 vs. M1) all indicated measurement equivalence (*p*-value = 0.014, ∆CFI = 0.000, ∆RMSEA = 0.000, ∆SRMR = 0.002) (Table 4).

As shown in the correlation matrix in Table 5, the GDT score was significantly correlated with scores on the GADIS-A CBS subscale (*r* = 0.72) and NC subscale (*r* = 0.66), IGDS9-SF (*r* = 0.71), DASS-21 (*r* = 0.17), BSMAS (*r* = 0.09), and SABAS (*r* = 0.13). Moreover, the score on the GADIS-A CBS subscale was significantly correlated with scores on the GADIS-A NC subscale (*r* = 0.84), IGDS9-SF (*r* = 0.74), DASS-21 (*r* = 0.16), BSMAS (*r* = 0.16), and SABAS (*r* = 0.24). Finally, the GADIS-A NC subscale was significantly correlated with scores on the IGDS9-SF (*r* = 0.72), DASS-21 (*r* = 0.17), BSMAS (*r* = 0.21), and SABAS (*r* = 0.24).

**Table 5:** Pearson correlations among the observed variables

	*r* (*p*-value)						
	1	2	3	4	5	6	7
1. GDT total score	1.00						
2. GADIS-A CBS score	0.72 (< .001)	1.00					
3. GADIS-A NC score	0.66 (< .001)	0.84 (< .001)	1.00				
4. IGDS9-SF total score	0.71 (< .001)	0.74 (< .001)	0.72 (< .001)	1.00			
5. DASS-21 total score	0.17 (< .001)	0.16 (< .001)	0.17 (< .001)	0.26 (< .001)	1.00		
6. BSMAS total score	0.09 (.031)	0.16 (< .001)	0.21 (< .001)	0.24 (< .001)	0.42 (< .001)	1.00	
7. SABAS total score	0.13 (.001)	0.24 (< .001)	0.24 (< .001)	0.29 (< .001)	0.42 (< .001)	0.63 (< .001)	1.00

*GDT* Gaming Disorder Test, *GADIS-A* Gaming Disorder Scale for Adolescents, *GADIS-A_CBS* Subscale of cognitive behavioral symptoms in GADIS-A, *GADIS-A_NC* Subscale of negative consequences in GADIS-A, *IGDS9-SF* Internet Gaming Disorder Scale–Short-Form, *DASS-21* Depression Anxiety Stress Scale-21, *BSMAS* Bergen Social Media Addiction Scale, *SABAS* Smartphone Application Based Addiction Scale

## Discussion

The present study translated the GDT and GADIS-A from English to Vietnamese for psychometric testing. More specifically, among Vietnamese students, the present study assessed (i) factor structures of the GDT and GADIS-A; (ii) invariance of the factor structure for both the GDT and GADIS-A; and (iii) convergent and divergent validity of the GDT and GADIS-A. In general, the CFA results indicated that model fit was acceptable for both the GDT (one-factor structure) and the GADIS-A (two-factor structure) confirming H_1_. Moreover, both scales showed excellent internal consistency among Vietnamese university students. According to measurement invariance analysis, the two-factor structure of GADIS-A was fully invariant whereas the one-factor structure of GDT was partially invariant across gender (males vs. females) and time spent on gaming (< 2 h daily vs. ≥ 2 h daily). Moreover, the GDT and GADIS-A showed excellent concurrent and divergent validity with standardized instruments to assess social medial addiction, smartphone addiction and psychological distress (confirming H_4_).

Consistent with the results of previous studies (Chen et al., 2023; Ghazi et al., 2024; Nazari et al., 2022; Paschke et al., 2020; Pontes et al., 2021; Wu et al., 2023), it was found that the construct validity findings supported the one-factor structure of the GDT and the two-factor structure of the GADIS-A. Moreover, both instruments had excellent internal consistency among young adults which was comparable with previous psychometric testing of the GDT and GADIS-A (Chen et al., 2023; Ghazi et al., 2024; Wu et al., 2023). Although the original validation study of the GADIS-A was specifically developed to assess adolescents’ GD (Paschke et al., 2020), the present study confirmed that the Vietnamese version of GADIS-A showed high levels of scale reliability across a different age group (i.e., young adults) with similar findings to recent Chinese and Malaysian research psychometrically testing the GADIS-A among emerging adults (Chen et al., 2023; Ghazi et al., 2024; Wu et al., 2023).

Regarding the results of demographic factors (Table 1), the study participants had a mean age of 21.09 years (ranging between 19 and 33 years), demonstrating that the GADIS-A is a feasible instrument to assess GD among emerging adults. Moreover, literature suggests that using the GADIS-A might help understand gaming-related behaviors across age groups (adolescents and young adults) (Ghazi et al., 2024; Wu et al., 2023). Utilizing the same instrument to assess GD across age groups could further help in the conducting of longitudinal studies across the lifespan from early adolescence to young adulthood (Ghazi et al., 2024). In sum, the present study indicated that both Vietnamese versions of the GDT and GADIS-A are useful instruments for assessing GD and can be used by Vietnamese healthcare providers and researchers to screen for and assess GD among young adults.

The findings of the present study partially supported H_2_. More specifically, the GADIS-A was found to be fully invariant whereas the GDT was found to be only partially invariant across gender (males vs. females) and time spent on gaming (< 2 h daily vs. ≥ 2 h daily). The GDT’s changes in fit indices (i.e., ΔRMSEA, ΔSRMR) were slightly higher than cutoff values of fit indices supporting measurement invariance, especially among one of the factor-loading-constrained models (i.e., M1 vs. M0) (Table 4). Literature has indicated that RMSEA and SRMR can be affected by small degrees of freedom and large sample sizes. Therefore, it has been suggested using ΔCFI as the primary criterion to support measurement invariance (Meade et al., 2008). However, the present study found that although the GDT showed poor model fit and violated the invariance testing recommendation across subgroups, all the values were acceptable using the model fit indices (i.e., CFI > 0.95, RMSEA < 0.05, and SRMR < 0.05). Therefore, Vietnamese version of the GDT and GADIS-A has the potential to assess GD across subgroups (i.e., gender and daily time spent on gaming) among young adults. However, further examination with a larger sample and more diverse age groups are required to test the validity of the factor structure of the GDT and GADIS-A.

The findings of the present study fully supported H_3_. GDT scores were significantly correlated with scores on the GADIS-A CBS subscale (*r* = 0.72) and NC subscale (*r* = 0.66). These findings concur with previous research reporting strong correlations between the GDT and GADIS-A (both CBS and NC subscales) (Chen et al., 2023; Ghazi et al., 2024; Wu et al., 2023). A previous study found that both the GDT and GADIS-A, which both use the ICD-11 GD criteria, are valid instruments (Karhulahti et al., 2023). Similarly, the present results indicated that both instruments appear to accurately screen and assess symptoms related to gaming problems using the ICD-11 diagnostic criteria for GD.

The present study’s results were also consistent with the prior psychometric GDT and GADIS-A studies in that the scores on the GDT and GADIS-A (both CBS and NC subscales) were significantly correlated with scores on the IGDS9-SF, BSMAS, and DASS-21 (Chen et al., 2023; Evren et al., 2020; Ghazi et al., 2024; Paschke et al., 2020; Pontes et al., 2021; Wu et al., 2023). Table 4 shows that IGDS9-SF score was highly associated with scores on both the GDT (*r* = 0.71) and GADIS-A (*r* = 0.72–0.74). These findings concur with previous studies, including findings from Chinese (*r* = 0.66–0.67) and Malaysian (*r* = 0.70–0.79) studies (Chen et al., 2023; Ghazi et al., 2024; Wu et al., 2023). Therefore, studies have noted that although GDT and GADIS-A were developed using the conceptualization of GD in the ICD-11, they share some similarity to the way IGD is defined in the DSM-5 (Pontes et al., 2021; Wu et al., 2023). Therefore, the GDT and GADIS-A have convergent validity with IGDS9-SF.

Consistent with past findings (Ghazi et al., 2024; Wu et al., 2023), the GDT and GADIS-A scores in the present study were weakly to moderately associated with BSMAS and DASS-21 scores. Moreover, the present study found that the GDT and GADIS-A scores were weakly associated with SABAS scores. The weak to moderate associations (rather than strong associations) may be explained by the different concepts assessed by the different instruments (i.e., gaming disorder in the GDT and GADIS-A; social medial addiction in the BSMAS; smartphone addiction in the SABAS; and psychological distress in the DASS-21). Therefore, the GDT and GADIS-A have divergent and concurrent validity with BSMAS, SABAS, and DASS-21.

In addition, the present study found that there was a significant correlation between DASS-21 and both the GDT and GADIS-A. This finding was comparable to a previous study (Wu et al., 2023). However, the present study’s findings showed that the DASS-21 had relatively weak correlation with the Vietnamese GDT (*r* = 0.17) and GADIS-A (*r* = 0.16–0.17) whereas the DASS-21 had a moderate correlation with the Chinese GDT (*r* = 0.35) and GADIS-A (*r* = 0.33 – 0.40) (Wu et al., 2023). A previous Vietnamese study indicated that young males who played multiplayer online role-playing games (MMORPGs) had greater scores on psychological distress (Son et al., 2013). The present study’s results might be different from previous findings because the majority of the participants in the present study were females (63.4%). Moreover, the extant literature has indicated that males tend to engage more regularly in online gaming more than females (Mari et al., 2023; Ruckwongpatr et al., 2024). Therefore, most of the participants in the present study might have engaged in other online activities (e.g., social media use) and be less likely to have gaming problems. However, the present study suggests more investigation between GD and psychological distress among Vietnamese populations is needed to corroborate the present findings.

Some limitations of the present study must be considered when interpreting the findings. First, self-report scales were used in the present study, therefore the findings may have been affected by recall or social desirability biases. Second, due to the cross-sectional study design, the analysis was unable to determine any cause-effect relationships between the study's variables. Third, the present study comprised a gender-imbalanced sample (63.4% females and 36.6% males) which may have impacted the study findings. Finally, the participants were Vietnamese university students who were recruited via convenience sampling. However, as aforementioned, the present study purposely sampled university students given that emerging adults are among the most vulnerable to GD, and convenience sampling of university students provides one of the easiest ways to access this cohort. However, university students are not nationally representative and therefore the psychometric properties of the Vietnamese versions of the GDT and GADIS-A will need further testing (e.g., measurement invariance) on other cohorts (e.g., adolescents, older adolescents) as well as testing with more nationally representative samples to increase the generalizability of the findings presented here. Despite, the non-generalizability of the sample, the present study is the first study in Vietnam to present primary evidence of the psychometric properties of two GD instruments (i.e., GDT and GADIS-A) based on ICD-11 diagnostic criteria. The findings demonstrated that both instruments are valid and reliable in assessing GD among Vietnamese university students which might be useful for healthcare workers to utilize these instruments to prevent GD symptoms in clinical management and outcome.

## Conclusion

The present study's findings provide psychometric evidence for the Vietnamese GDT and GADIS-A among young adults. It was found that both GDT and GADIS-A are valid and reliable measures indicated by the promising psychometric properties that assessed levels of GD symptoms using ICD-11 criteria. The Vietnamese version of GDT and GADIS-A may serve as useful instruments to assess problematic gaming in Vietnam. This will help increase research on GD in the country as well as help in the design of prevention and intervention programs to reduce the negative health and psychosocial consequences. Research is needed to further evaluate the psychometric properties of the GDT and GADIS-A among various age groups both in Vietnam and in other countries and cultures.

## Abbreviations

GDT: Gaming Disorder Test
GADIS-A: Gaming Disorder Scale for Adolescents
CFA: Confirmatory factor analysis
GD: Gaming disorder
WHO: World Health Organization
ICD-11: International Classification of Diseases, 11th Revision
APA: American Psychiatric Association
IGD: Internet gaming disorder
DSM-5: Fifth edition of Diagnostic and Statistical Manual of Mental Disorders
IGDT-20: 20-Item Internet Gaming Disorder Test
BSMAS: Bergen Social Media Addiction Scale
SABAS: Smartphone Application-Based Addiction Scale
IGDS9-SF: Internet Gaming Disorder Scale-Short Form
DASS-21: Depression Anxiety and Stress Scale -21
DWLS: Diagonally weighted least squares
SRMR: Standardized root mean square residual
RMSEA: Root mean square error of approximation
TLI: Tucker-Lewis index
CFI: Comparative fit index
MGCFA: Multi-group confirmatory factor analysis
